# A predictive risk‐scoring model for survival prognosis of multiple myeloma based on gain/amplification of 1q21: Experience in a tertiary hospital in South‐Western China

**DOI:** 10.1002/cam4.70193

**Published:** 2024-09-05

**Authors:** Yanqiu Xiong, Shanshan Liang, Wenjiao Tang, Li Zhang, Yuhuan Zheng, Ling Pan, Ting Niu

**Affiliations:** ^1^ Department of Hematology Insitute of Hematology, West China Hospital, Sichuan University Chengdu China; ^2^ Department of Hematology Clincal Medical College & Affiliated Hospital of Chengdu University Chengdu China; ^3^ Department of Laboratory Medicine West China Hospital, Sichuan University Chengdu China

**Keywords:** 1q21, amplification, gain, high risk, myeloma, survival

## Abstract

**Background:**

Chromosomal 1q gains and amplifications (+1q21) are frequently observed in patients with newly diagnosed multiple myeloma (NDMM). However, the interpretation of the high‐risk (HR) prognostic implications stemming from 1q21 abnormalities remain challenging to implement effectively.

**Methods:**

In a comprehensive analysis of 367 consecutive patients with symptomatic MM, we assessed the prognostic significance of +1q21 using FISH with a threshold of 7.4%. The patient cohort was randomly divided into a training set (66.5%, *n* = 244) and a validation set (33.5%, *n* = 133). A multivariate Cox regression analysis was conducted to identify significant prognostic factors associated with PFS. Weight scores were assigned to each risk factor based on the β‐value of the corresponding regression coefficient. A predictive risk‐scoring model involving +1q21 was then developed, utilizing the total score derived from these weight scores. The model's discriminative ability was evaluated using the AUC in both the training and validation sets. Finally, we compared the performance of the +1q21‐involved risk with the established R‐ISS and R2‐ISS models.

**Results:**

Upon initial diagnosis, 159 patients (43.32%) exhibited +1q21, with 94 (59.11%) having three copies, referred to as Gain(1q21), and 65 (40.89%) possessing four or more copies, referred to as Amp (1q21). Both were significantly linked to a reduced PFS in myeloma (*p* < 0.05), which could be effectively mitigated by ASCT. The +1q21‐involved risk model, with an AUC of 0.697 in the training set and 0.725 in the validation set, was constructed including Gain(1q21), Amp(1q21), no‐ASCT, and TP53 deletion. This model, termed the ultra‐high‐risk (UHR) model, demonstrated superior performance in predicting shorter PFS compared to the R‐ISS stage 3 and R2‐ISS stage 4.

**Conclusion:**

The UHR model, which integrates the presence of +1q21 with no‐ASCT and TP53 deletion, is designed to identify the early relapse subgroup among patients with +1q21 in NDMM.

## 1 INTRODUCTION

Multiple myeloma (MM) is distinguished by the abnormal proliferation of monoclonal plasma cells within the bone marrow,^1^ representing a complex disease with a spectrum of genetic alterations and significant prognostic heterogeneity.^2^, ^3^ Cytogenetic abnormalities (CAs) are pivotal in the risk stratification of newly diagnosed multiple myeloma (NDMM), with high‐risk cytogenetic abnormalities (HRCAs) including chromosomal 1q gains and amplifications (+1q21), deletion of 17p, and translocations t(4;14), t(14;16), and t(14;20).^4^ Previously, the Revised International Staging System (R‐ISS) accounts for only three CAs,^5^ namely, translocations t(4;14), t(14;16), and deletion del(17p). It is crucial to recognize that other abnormalities, such as +1q21, are also correlated with inferior prognostic outcomes.^2^, ^6^, ^7^ Currently, the Second Revision of the International Staging System (R2‐ISS) has expanded the scope to include +1q21,^8^ frequently observed as the most prevalent secondary genetic event in approximately 30%–40% of patients at initial diagnosis.^7^, ^9^, ^10^ It is often implicated in jumping translocations involving a segment or the entire long arm of chromosome 1, which is associated with tumor progression and advanced disease.^11^, ^12^, ^13^

Prior to the establishment of the R2‐ISS staging system, there was debate surrounding the role of +1q21 as an independent prognostic indicator.^14^ The prognostic significance of copy number abnormalities of 1q21 is similarly contentious; some studies suggest that copy number alone does not enhance prognostic value,^15^, ^16^ while others propose that an elevated copy number, particularly when four or more copies of 1q21 are present, is associated with the most adverse prognosis.^5^, ^17^, ^18^ Specifically, within the context of ISS stage 3, amplification of CKS1B (located on 1q21) with a copy number of four or more has been identified as a hallmark of an ultra‐high‐risk (UHR) population in myeloma.^11^ Besides, in clinical practice, the interpretation of the high‐risk (HR) prognostic implications stemming from high‐frequency 1q21 abnormalities remains challenging to implement effectively.

The primary objective of this study is to develop a predictive risk model, firmly established on the thorough analysis of chromosome 1q21, with the aim of distinguishing the HR and UHR subgroups among patients with +1q21 in NDMM.

## 2 METHODS

### 2.1 Patients and treatments

In this study, we enrolled 367 patients with NDMM admitted to West China Hospital of Sichuan University between February 1, 2016, and March 31, 2023, excluding those with incomplete cytogenetic data. The diagnosis and response assessment for symptomatic MM adhered to the criteria set by the International Myeloma Working Group (IMWG).^2^ Follow‐up for all patients continued until December 1, 2023. The baseline characteristics encompassed demographic specifics like age and gender, alongside laboratory indicators such as hemoglobin levels, blood platelet counts, lactate dehydrogenase (LDH) concentrations, serum creatinine levels, and cytogenetic evaluations utilizing G‐banding karyotype and fluorescence in situ hybridization (FISH) assays. Additionally, we gathered thorough data pertaining to the diagnosis date, treatment commencement, induction therapy， upfront autologous stem cell transplantation (ASCT), consolidation and/or maintenance therapy, the optimal response to induction therapy and transplantation, and pivotal dates indicating disease progression and mortality. Induction schemes include proteasome inhibitor‐based regimens (BD, BCD, etc.), immunoregulator‐based regimens (RD, BRD, etc.), and other treatment options (like CD).

### 2.2 Using dynamic threshold setting in the FISH method

The procedure entails the extraction of 15–20 mm of bone marrow, anticoagulated with heparin, followed by centrifugation to isolate the mononuclear cells. Subsequently, micromagnetic beads conjugated with CD138 antibodies are introduced to selectively bind to specific cells. Once the sorting buffer is added, the cells undergo rinsing and separation via a column chromatography technique. The purified plasma cells are then fixed and cryopreserved. In parallel, a panel of fluorescent probes targeting 1q21, RB1, D13S319, TP53, IGH/FGFR3, IGH/CCND1, IGH/MAF, IGH/MAFB, and IGH/CCND3 loci is incorporated into the suspension for labeling purposes. This is succeeded by denaturation, hybridization, and staining with 4′,6‐diamidino‐2‐phenylindole (DAPI) to facilitate microscopic analysis.

Dynamic threshold determination was executed by concurrently analyzing 20 normal human specimens to quantify the nuclei exhibiting diverse signaling patterns. The gain and/or amplification of the 1q21 copy number were delineated as a positive +1q21 status when present in no less than 7.4% of the neoplastic cell population.

### 2.3 Model construction

For the development of the +1q21‐involved risk model, the patient cohort was randomly divided, allocating two‐thirds to the training set and the remaining one‐third to the validation set. The training set was instrumental in identifying the predictors for early relapse, whereas the validation set was used to evaluate the model's performance. Univariate analyses within the training set pinpointed independent risk factors, with those significant at the *p* < 0.100 level being selected for inclusion in the subsequent Cox regression model as covariates. A comprehensive multivariate Cox regression analysis was then performed to assess the prognostic significance of these factors, quantified by hazard ratios (HRs) and their corresponding 95% confidence intervals (CIs). Each risk factor was assigned a weight score derived from the β‐value of the regression coefficient, which was then utilized to formulate the predictive model based on the aggregated total scores. To appraise the model's discriminative power, receiver operating characteristic (ROC) curve analysis was applied to censored survival data, yielding the area under the curve (AUC) and determining the optimal cut‐off values through maximizing the Youden index, defined as sensitivity plus specificity minus 1. An AUC value of 1.0 signifies an ideal predictive model, whereas a value of 0.5 equates to the probability of correct prediction by random chance.

### 2.4 Statistical analysis

Progression‐free survival (PFS) was defined as the interval from the initial diagnosis to the first occurrence of death, disease progression, or the last follow‐up contact. Tailoring the statistical analysis, the selection of the chi‐square test, *t*‐test, or Fisher's exact test was made based on the specific characteristics of the data. Subsequently, the Kaplan–Meier method was used to construct survival curves, and the log‐rank test was employed to compare the survival distributions. A *p*‐value threshold of less than 0.050 was set to denote statistical significance. The statistical analysis was performed using R software 4.0, incorporating the ‘rms’, ‘survival’, and ‘survival ROC package’,^19^ as well as Prism 9.0, SPSS 26.0, and R software 4.0. All P‐values were two‐ tailed.

## 3 RESULTS

### 3.1 MM patients with +1q21 aberration demonstrated higher prevalence of severe anemia, advanced disease stages, and elevated high‐risk genetic abnormalities

The analysis revealed that 159 of the 367 patients (43.32%) presented with either a gain or amplification in the 1q21 chromosomal region. Of these, 59.11% (94) carried three copies, referred to as Gain(1q21); while 40.89% (65) had four or more copies of the 1q21 segment, referred to as Amp (1q21). A comparative analysis of baseline clinical data between these two subgroups, presented in Table 1, revealed no statistically significant differences.

**TABLE 1:** Baseline of clinical characteristics of NDMM patients.

	1q21 (−) *N* = 208 (56.68%)	1q21 (+) *N* = 159 (43.32%)	OR (95% CI)	*p* value	Gain (1q21) *N* = 94(59.11%)	Amp (1q21) *N* = 65(40.89%)	OR (95% CI)	*p* value
Age (years)	58 ± 10.6	60 ± 10.7	NA	0.191	59 ± 11.5	60 ± 9.5	NA	0.276
Sex (male)	119/208 (57.21%)	87/159 (54.71%)	1.107 (0.730–1.677)	0.633	52/94 (55.3152%)	36/65 (59.38%)	0.997 (0.528–1.844)	0.993
High LDH	44/208 (21.15%)	44/159 (27.67%)	1.426 (0.881–2.307)	0.147	25/94 (26.59%)	19/65 (29.23%)	1.140 (0.564–2.304)	0.715
HB (g/L)	105 ± 28.7	92 ± 27.5	NA	<0.001	95 ± 28.1	87 ± 25.9	NA	0.053
PLT (10^9^/L)	172 ± 67.3	165 ± 73.82	NA	0.365	165 ± 69.3	164 ± 80.4	NA	0.932
RI	63/208 (30.29%)	54/159 (33.96%)	1.184 (0.761–1.841)	0.454	30/94 (31.91%)	25/65 (38.36%)	1.333 (0.688–2.584)	0.394
EMD	34/208 (16.35%)	32/159 (20.13%)	1.289 (0.756–2.200)	0.350	20/94 (21.28%)	12/65 (18.46%)	0.838 (0.377–1.860)	0.663
DS			NA	0.001			NA	0.06
I II III	51/208 (24.52%) 32/208 (15.38%) 125/208 (60.10%)	20/159 (12.58%) 14/159 (8.81%) 125/159 (78.61%)			16/94 (17.02%) 10/94 (10.63%) 68/94 (72.34%)	4/65 (6.15%) 4/65 (6.15%) 57/65 (87.69%)		0
ISS			NA	<0.001			NA	0.241
I II III	88/208 (42.31%) 56/208 (26.92%) 64/208 (30.77%)	34/159 (21.38%) 52/159 (32.70%) 73/159 (45.91%)			22/94 (23.40%) 34/94 (36.17%) 38/94 (40.43%)	12/65 (18.46%) 18/65 (27.69%) 35/65 (53.85%)		
R‐ISS			NA	<0.001			NA	0.535
I II III	79/208 (37.98%) 104/208 (50.00%) 25/208 (12.02%)	19/159 (11.95%) 90/159 (56.60%) 50/159 (31.45%)			13/94 (13.83%) 54/94 (57.45%) 27/94 (28.72%)	6/65 (9.23%) 36/65 (55.38%) 23/65 (35.39%)		
R2‐ISS I II III IV	76/208 (36.54%) 48/208 (23.08%) 81/208 (38.94%) 3/208 (1.44%)	1/159 (0.63%) 18/159 (11.32%) 93/159 (58.49%) 47/159 (29.56%)	NA	<0.001	1/94 (1.06%) 13/94 (13.83%) 56/94 (59.57%) 24/94 (25.54%)	0/65 (0.00%) 5/65 (7.69%) 37/65 (56.92%) 23/65 (35.39%)	NA	NA
TP53 deletion	13/208 (6.25%)	25/159 (15.72%)	2.799 (1.382–5.666)	0.003	16/94 (17.02%)	9/65 (13.84%)	0.783 (0.323–1.900)	0.589
t (11;14)	15/208 (7.21%)	13/159 (8.18%)	1.146 (0.529–2.482)	0.730	9/94 (9.57%)	4/65 (6.15%)	0.619 (0.182–2.104)	0.562
t (4;14)	9/208 (4.32%)	28/159 (17.61%)	4.726 (2.161–10.338)	<0.001	14/94 (14.89%)	14/65 (21.53%)	1.569 (0.691–3.561)	0.280
t (14;16)	0	6/159 (3.73%)	NA	NA	2/94 (2.13%)	4/65 (6.15%)	3.016 (0.536–16.980)	0.227
t (6;14)	3/208 (1.44%)	4/159 (2.52%)	1.763 (0.389–7.994)	0.456	4/94 (4.26%)	0	NA	NA
t (14;20)	0	0	NA	NA	0	0	NA	NA
High‐t (14)	8/208 (3.85%)	33/159 (20.75%)	6.548 (2.931–14.629)	<0.001	15/94 (15.96%)	18/65 (27.69%)	2.017 (0.930–4.376)	0.073
RB1 deletion	37/208 (17.78%)	81/159 (50.94%)	4.799 (2.993–7.696)	<0.001	51/94 (54.25%)	30/65 (46.15%)	0.723 (0.383–1.363)	0.315
D13S319 deletion	37/209 (17.78%)	81/159 (50.94%)	4.799 (2.993–7.696)	<0.001	51/94 (54.25%)	30/65 (46.15%)	0.723 (0.383–1.363)	0.315
Karyotype abnormalities	10/195 (5.13%)	29/150 (19.33%)	86/142(60.56%) 12/142 (8.45%) 44/142 (30.98%)		16/90 (17.78%)	13/60 (21.67%)	1.279 (0.565–2.899)	0.555
First‐line therapy PI‐based IMiD‐based PI+ IMiD‐based	111/174 (63.79%) 15/174 (8.62%) 48/174 (27.59%)			0.801	46/81 (56.79%) 6/81 (7.41%) 29/81 (35.80%)	40/61 (65.57%) 6/61 (9.84%) 15/61 (24.59%)		0.350

*Note*: 1q21(−), patients without 1q gain/amp; 1q21(+), patients with 1q gain/amp; Gain(1q21), 1q21 with copies =3; Amp(1q21), 1q21 with copies ≥4; high‐t^14^ according to mSMART 3.0 includes t^4^; 14, t (14; 16) or t(14; 20); EMD, The values for hemoglobin, age as well as platelets indicate mean ± SD.

Abbreviations: CI, confidence interval; EMD, extramedullary myeloma disease; Hb, hemoglobin; IMiD, immunomoduator; ISS, international stage system; NA, not applicable; OR, odds ratio; PI, protease inhibitor; PLT, platelet; RI, *r*enal insufficiency; R‐ISS, revised international stage System; R2‐ISS, second revision of the international staging system.

In contrast to patients without the +1q21 aberration, as outlined in Table 1, those with +1q21 exhibited a significantly higher prevalence of severe anemia (*p* < 0.001) and more advanced disease stages, including a higher International Staging System (ISS) score (*p* < 0.001), R‐ISS stage (*p* < 0.001), and R2‐ISS stage (*p* < 0.001). Furthermore, they displayed an increased incidence of HRCAs, such as TP53 deletion (*p* = 0.003) and t(4;14) translocation (*p* < 0.001). After incorporating chromosome 14 translocations, specifically t(4;14), t(14;16), and t(14;20), which constitute hallmarks of the high‐risk category in the mSMART 3.0 criteria,^20^ it was also determined that these high‐risk Chromosome 14 translocations (high‐t(14)) were significantly associated with the +1q21 aberration (*p*<0.001). Additionally, the presence of +1q21 was more frequently observed in conjunction with deletions of RB1 and D13S319, as well as complex karyotypes (*p* < 0.001).

### 3.2 Gain(1q21)‐combined and Amp(1q21)‐involved mutations were associated with shorter PFS in myeloma

**FIGURE 1:**
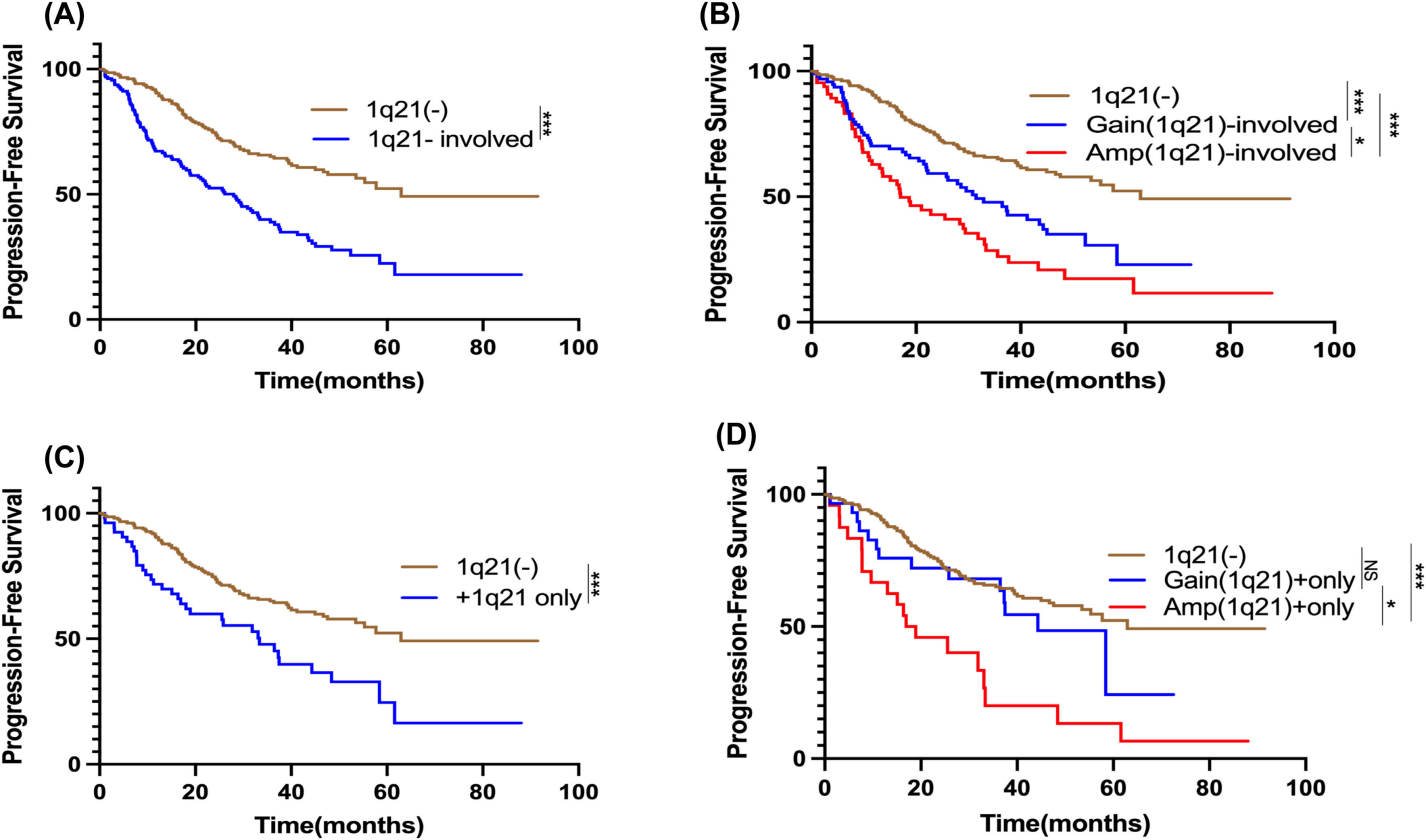
Impact of +1q21 on the Outcomes of 367 Patients with Newly Diagnosed Multiple Myeloma (NDMM). (A) Kaplan–Meier estimates revealed a significant difference in median progression‐free survivals (mPFSs) between patients with and without +1q21 aberration, with medians of 27.93 and 62.90 months, respectively (*p*<0.001). (B) Among patients without +1q21, Gain(1q21), and Amp(1q21), the mPFSs showed distinct differences: 62.90 months for those without +1q21, 31.47 months for those with Gain(1q21), and 17.00 months for those with Amp(1q21). HRs indicated significant risks: Gain(1q21) vs. 1q21(‐), HR=1.957, *p*<0.001; Amp(1q21) vs. 1q21(‐), HR=2.955, *p*<0.001; and Amp(1q21) vs. Gain(1q21), HR=1.517, *p*=0.033. (C) A significant difference in mPFSs between thosewith isolated +1q21 aberration and without +1q21, as 62.90 versus 33.37 months (*p*<0.001). (D)Patients without +1q21 had a mPFS of 62.90 months, those with isolated Gain(1q21) had a mPFS of 44.33 months, and those with isolated Amp(1q21) had a mPFS of 17.92 months. HRs: Gain(1q21)+only vs. 1q21(‐), HR = 1.368, *p*=0.277; Amp(1q21)+only vs. 1q21(‐), HR = 3.188, *p*<0.001; Amp(1q21)+only vs. Gain(1q21)+only, HR =2.264, *p*=0.015. ***p<0.001, **p<0.01, *p<0.05, by two‐sided log‐rank test.

The median PFS were 41.20 (95%CIs, 31.15–51.24) months in the overallcohort. Patients with the +1q21 aberration experienced a notably reduced median PFS of 27.93 months, in contrast to the 62.90 months observed in the 1q21‐negative group (1q21(−)) (HR = 2.327, *p* < 0.001) (Figure 1A). Moreover, a stepwise decrease in median PFS was evident among patients with varying copy numbers of 1q21, with those having 2, 3, and ≥4 copies showing median PFS of 62.90, 31.47, and 17.00 months, respectively, highlighting a significant association with copy number and survival outcomes (Figure 1B).

When +1q21 was the sole cytogenetic abnormality, the PFS was also significantly shorter than that of the 1q21(−) group with 33.37 than 62.90 months (*p* < 0.001) (Figure 1C). Furthermore, a significant difference in PFS was observed between the subgroup exhibiting Amp(1q21) and 1q21(−) group, whereas the subgroup with sole Gain(1q21) did not exhibit a comparable distinction (Figure 1D).

### 3.3 Upfront ASCT conquered the negative prognosis of PFS for myeloma patients with +1q21

**FIGURE 2:**
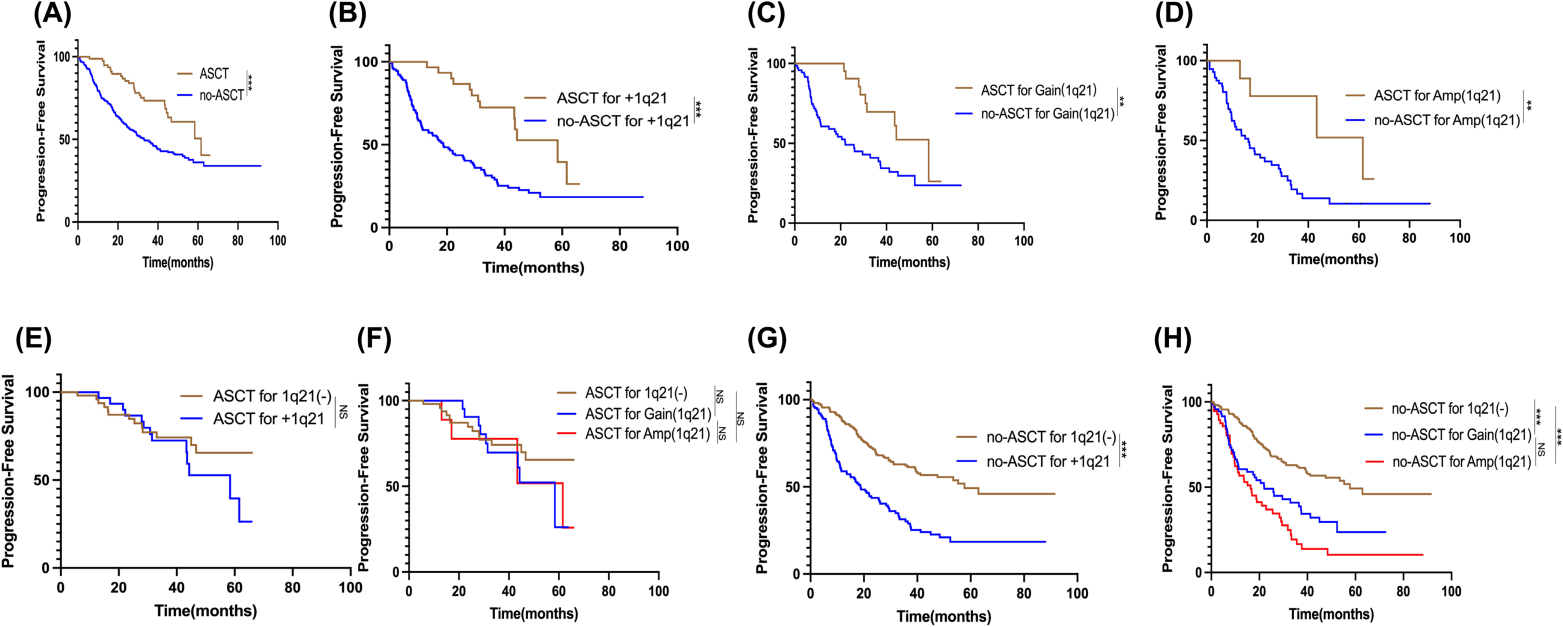
Effect of ASCT on outcomes in patients with 367 NDMM with or without +1q21 Aberration. (A) Kaplan‐Meier analysis showed a significant difference in PFS between patients who had ASCT and those who had not, with median PFS of 61.60 and 33.10 months, respectively (*p*<0.001). (B) Patients with +1q21 NDMM who received ASCT had a much longer PFS of 58.43 months compared to those who did not, with only 18.73 months (*p*<0.001). (C) Patients with Gain(1q21) had a median PFS of 58.43 months with ASCT compared to 22.00 months without ASCT (*p*=0.008). (D) Patients with Amp(1q21) who received ASCT had a median PFS of 61.60 months, compared to 16.37 months for those who did not receive ASCT (*p*=0.007). (E) In 82 NDMM patients undergoing ASCT, there was no significant difference in PFS between those with 1q21(+) and 1q21(‐) (*p* = 0.397). (F) In 82 NDMM patients undergoing ASCT, patients with 2，3 and ≥4 copies of 1q21 showed no significant differences in PFS with median values of NR, 58.43 and 61.60 months, respectively; HRs: Gain(1q21) vs. 1q21(‐), HR = 1.380, *p*=0.453; Amp(1q21) vs. 1q21(‐), HR = 1.414, *p*=0.533; Amp(1q21) vs. Gain(1q21), HR =0.941, *p*=0.917. (G) In 285 NDMM patients without ASCT, those with +1q21 aberration had a significantly shorter mPFS of 18.73 months compared to those without +1q21 aberration at 57.67 months (*p*<0.001). (H) In 285 NDMM patients without ASCT，PFS was shorter in the Gain(1q21) and Amp(1q21) groups compared to the 1q21(‐) group. There was no significant difference in PFS between the Gain(1q21) and Amp(1q21) groups. HRs: Gain(1q21) vs. 1q21(‐), HR = 2.202, *p*<0.001; Amp(1q21) vs. 1q21(‐), HR = 3.216, *p*<0.001; Amp(1q21) vs. Gain(1q21), HR =1.463, *p*=0.069. ***p<0.001, **p<0.01, *p<0.05, by two‐sided log‐rank test.

Only 22.34% (82 patients) underwent upfront ASCT in the overall cohort, with 78 cases being patients aged ≤65 years who received upfront ASCT. Comparing the group with ASCT to the group without ASCT, there was a significant benefit in terms of PFS, with respective median PFS of 61.60 and 33.10 months (HR = 2.174, *p<*0.001) (Figure 2A). And the PFS of patients with +1q21 was significantly improved from 18.73 months to 58.43 months by ASCT (HR = 2.966, *p<*0.001) (Figure 2B). With ASCT, the PFS of patients with Gain(1q21) was improved significantly from 22 months to 58.43 months (HR = 2.538, *p* = 0.008) (Figure 2C), as was the PFS for Amp(1q21) patients from 16.37 months to 61.60 months with ASCT (HR = 3.559, *p* = 0.007) (Figure 2D).

In the upfront ASCT cohort, no statistically significant difference in PFS was observed between the +1q21 and the 1q21(−) group, with median values of 58.43 months and not reached (NR) (HR = 1.387, *p =* 0.397) (Figure 2E); while in the no‐ASCT cohort, patients with +1q21 had a shorter PFS compared to those without +1q21 (18.73 to 57.67 months, HR = 2.573, *p<*0.001) (Figure 2G).

Further analysis revealed that in the upfront ASCT cohort, patients with 2，3 and ≥4 copies of 1q21 showed no significant differences in PFS with median values of NR, 58.43 and 61.60 months, respectively (Figure 2F). Meanwhile, in the no‐ASCT cohort, compared to the 1q21(−) group with a PFS of 57.67 months, the PFS were shorter in both the Gain(1q) group and the Amp(1q) group (22.00 months, HR = 2.202, *p<*0.001; 16.37 months, HR = 3.216, *p <* 0.001); while no significant difference of PFS was found between the latter two groups(Figure 2H).

### 3.4 A predictive risk model was developed integrating gain/amplification of 1q21, no‐ASCT and TP53 deletion

**FIGURE 3:**
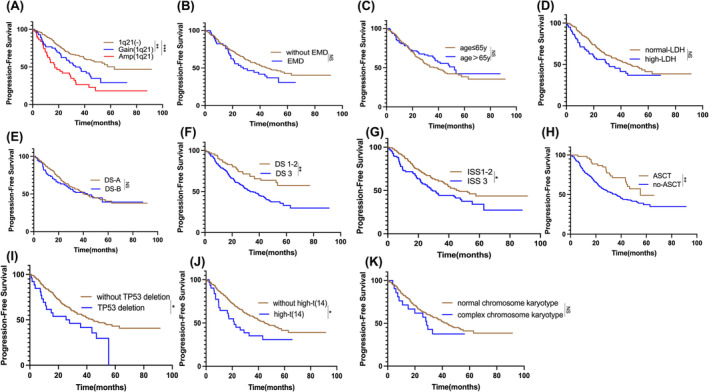
Effects of various factors on PFS in a cohort of 244 NDMM patients in the training set. (A) Kaplan‐Meier analysis showed significant differences in PFS between patients with Gain(1q21) and Amp(1q21) compared to those without 1q21 abnormalities: Gain(1q21) vs. 1q21(‐), *p*=0.006; Amp(1q21) vs. 1q21(‐), *p*<0.001. (B) Kaplan‐Meier analysis showed no significant difference in PFS between patients with and without EMD; *p*=0.121. (C) Kaplan‐Meier analysis showed no significant difference in PFS between patients age≤65y and those>65y; *p*=0.250. (D) Kaplan‐Meier analysis showed no significant difference in PFS between patients with normal‐LDH and with high‐LDH; *p*=0.095. (E) Kaplan‐Meier analysis showed no significant difference in PFS between patients with DS‐A and with DS‐B; *p*=0.748. (F) Kaplan‐Meier analysis showed significant difference in PFS between patients with DS 1‐2 and with DS 3; *p*=0.002; (G) Kaplan‐Meier analysis showed significant difference in PFS between patients with ISS 1‐2 and with ISS 3, *p*=0.033. (H) Kaplan‐Meier analysis showed significant difference in PFS between patients with ASCT and without ASCT, *p*=0.008; (I) Kaplan‐Meier analysis showed significant difference in PFS between patients with TP53 deletion and without TP53 deletion, *p*=0.024. (J) Kaplan‐Meier analysis showed significant difference in PFS between patients with high‐t(14) and without high‐t(14), *p*=0.015. (K) Kaplan‐Meier analysis showed no significant difference in PFS between patients with normal‐chromosome karyotype and with complex‐chromosome karyotype, *p*=0.336; *EMD*, Extramedullary Myeloma Disease; *DS‐A*, *no‐*renal insufficiency; *DS*‐*B*, renal insufficiency; high‐t(14) according to mSMART 3.0 Includes t(4; 14), t (14; 16) or t(14; 20). ***p<0.001, ^**^ *p*<0.01, *p<0.05, by two‐sided log‐rank test.

Clinical features in the training set were presented in Table 2, univariate analysis showed that Gain (1q21) and Amp (1q21), as well as ISS III stage, DS III stage, TP53 deletion, no‐ASCT and high‐t(14), were significant variables for PFS (Figure 3, Table 3). Factors with a significance level of *p* < 0.1 (Gain (1q21), Amp (1q21), ISS III stage, DS III stage, TP53 deletion, no‐ASCT, high‐t(14) and high LDH) were included as variables in the Cox regression model. Furthermore, multivariable analyses of PFS were conducted, and the results are listed in Table 3. The results showed that Gain (1q21) [HR = 1.751 (1.139–2.691), *p* = 0.011] and Amp (1q21) [HR = 2.624 (1.640–4.200), *p* < 0.001], as well as no‐ASCT [HR = 2.340 (1.434–3.820), *p* = 0.001] and TP53 deletion [HR = 2.118 (1.254–3.577), *p* = 0.005], were independent predictors for PFS (Table 3).

**TABLE 2:** Baseline characteristics of NDMM patients in the training and validation sets.

Training set (*N* = 244)			Validation set (*N* = 123)	
Factor	N0 (%)	Median (IQR)	N0 (%)	Median (IQR)
Age (years)		60(51–66)		57 (28–85)
Sex (male)	129/244 (52.87%)		77/123 (62.60%)	
High LDH	59/244 (24.18%)		29/123 (23.58%)	
HB (g/L)		99 (80–124)		91 (32–160)
PLT (10^9^/L)		160 (119–207)		167 (20–324)
RI	71/244 (29.10%)		46/123 (36.51%)	
EMD	44/244 (18.03%)		21/123 (17.07%)	
DS				
I II II	38/244 (15.57%) 32/244 (13.12%) 174/244 (71.31%)		33/123 (26.82%) 14/123 (11.38%) 76/123 (61.79%)	
ISS				
I II III	86/244 (35.25%) 74/244 (30.33%) 84/244 (34.42%)		36/123 (29.27%) 34/123 (27.64%) 53/123 (43.09%)	
R‐ISS				
I II III	70/244 (28.69%) 132/244 (54.10%) 42/244 (17.21%)		28/123 (22.76%) 62/123 (50.41%) 33/123 (26.83%)	
R2‐ISS				
I II III IV	58/244 (23.77%) 43/244 (17.62%) 112/244 (45.90%) 31/244 (12.70%)		18/123 (14.63%) 23/123 (18.70%) 62/123 (50.41%) 19/123 (15.45%)	
TP53 deletion	26/244 (10.66%)		12/123 (9.76%)	
High‐t (14)	38/244 (15.66%)		10/123 (8.13%)	
RB1 deletion	78/244 (31.97%)		40/123 (32.52%)	
D13S319 deletion	78/244 (31.97%)		40/123 (32.52%)	
Karyotype abnormalities	20/228 (8.78%)		19/117 (16.24%)	
First‐line therapy				
PI‐based IMiD‐based PI + IMiD‐based	153/203 (75.37%) 16/203 (7.88%) 34/203 (16.75%)		44/110 (40.00%) 11/110 (10.00%) 58/110 (52.72%)	

*Note*: high‐t(14) according to mSMART 3.0 includes t(4;14), t (14; 16) or t (14; 20); PI, Protease inhibitor; IMiD, Immunomoduator.

Abbreviations: EMD, Extramedullary Myeloma Disease; IQR, Interquartile range; RI, Renal insufficiency.

**TABLE 3:** Univariate and multivariate outcome analysis in training set; score calculated from the weight scores.

Univariate analysis				Multivariate analysis			
		*p* value	HR (95% CI)	*β*	*p* value	HR (95% CI)	Score (β*10)
1q21 copy	Gain(1q21) Amp(1q21)	0.006 <0.001	1.900 (1.201–3.004) 2.714 (1.576–4.673)	0.560 0.965	0.011 <0.001	1.751 (1.139–2.691) 2.624 (1.640–4.200)	5.6 9.7
ASCT	No	0.008	1.868 (1.268–2.753)	0.850	0.001	2.340 (1.434–3.820)	8.5
TP53 deletion	Yes	0.024	1.756 (0.945–3.264)	0.751	0.005	2.118 (1.254–3.577)	7.5
High‐t(14)	Yes	0.015	1.661 (1.003–2.751)	0.247	0.346	1.280 (0.766–2.138)	‐
LDH	High	0.095	1.383 (0.912–2.097)	0.341	0.089	1.406 (0.946–2.084)	‐
DS	III	0.002	1.951 (1.360–2.798)	0.394	0.099	1.483 (0.929–2.367)	‐
ISS	III	0.033	1.455 (1.009–2.100)	−0.095	0.628	0.910 (0.620–1.334)	‐
Karyotype abnormalities	Yes	0.336	1.324 (0.695–2.519)	‐	‐	‐	‐
EMD	Yes	0.121	1.383 (0.877–2.179)	‐	‐	‐	‐
DS	B	0.748	1.063 (0.728–1.551)	‐	‐	‐	‐
Age	>65y	0.250	0.801 (0.577–1.151)	‐	‐	‐	‐

*Note*: 1q21(−), patients without 1q gain/amp; 1q21(+), patients with 1q gain/amp; Gain (1q21), 1q21 with copies =3; Amp(1q21), 1q21 with copies ≥4; high‐t(14) according to mSMART 3.0 includes t(4; 14), t (14; 16) or t (14; 20).

Abbreviations: DS‐A, no‐ renal insufficiency; DS‐B, renal insufficiency; EMD, extramedullary myeloma disease.

**FIGURE 4:**
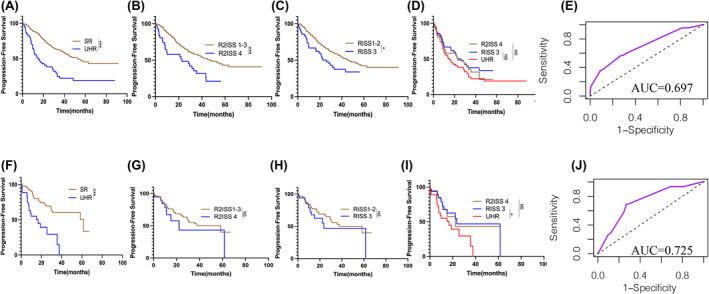
The +1q21‐involved in the establishment and verification of UHR Model and the comparison of this model with RISS staging and R2ISS staging in the training set and the validation set. (A) Kaplan‐Meier estimates revealed significant differences in PFS between the SR (standard risk group) and UHR (ultra‐high‐risk group) in the training set (53.47 months vs. 15.00 months, *p*<0.001); (B) Kaplan‐Meier estimates revealed significant differences in PFS between the R2ISS 1‐3 and R2ISS 4 in the training set (46.67 months vs. 22.80 months, *p*<0.001); (C) Kaplan‐Meier estimates revealed significant differences in PFS between the RISS 1‐2 and RISS 3 in the training set (45.03 months vs. 24.45 months, *p*=0.019); (D) Kaplan‐Meier estimates the UHR group had a shorter median PFS compared to the R‐ISS 3 and the R2ISS 4 in the training set; (15.00 months vs. 24.45 months *p*=0.169; 15.00 months vs. 24.45 months, *p*=0.621); (E) ROC curves of the +1q21‐involved UHR model in the training set. (F) Kaplan‐Meier estimates revealed significant differences in PFS between the SR and UHR in the validation set (61.60 months vs. 16.73months, *p*＜0.001); (G) Kaplan‐Meier estimates revealed significant differences in PFS between the R2ISS 1‐3 and R2ISS 4 in the validation set (58.47 months vs. 22.27 months, P=0.236); (H) Kaplan‐Meier estimates revealed significant differences in PFS between the RISS 1‐2 and RISS 3 in the validation set (37.70 months vs. 23.57 months, *p*=0.281); (I) Kaplan‐Meier estimates the UHR group had a shorter median PFS compared to the RISS 3 and the R2ISS 4 in the validation set; (16.73 months vs. 22.27 months *p*=0.164; 16.73 months vs. 23.57 months, *p*=0.045); (J) ROC curves of the +1q21‐involved UHR model in the validation set. ***p<0.001, **p<0.01, *p<0.05, by two‐sided log‐rank test.

Subsequently, a predictive risk‐scoring model was developed using the total score calculated from the weight scores (Table 3). Based on the scores obtained from the algorithm with total score of 31.30 points, patients were classified according to the median scores into the group <15.60 points and the group ≥15.60 points. In the latter group, 57 patients had a median PFS of 15.00 months, significantly shorter than 53.47 months in the former group harboring 197 patients with NDMM (HR = 2.663, *p*<0.001) (Figure 4A). Because the PFS of the group ≥15.60 points quite closed to the time of early relapse in clinical practice, the +1q21‐involved model with the AUC value of 0.697 was abbreviated as the UHR model in this study(Figure 4E).

In the validation set of 123 patients (Table 2), the AUC value of the +1q21‐involved UHR model was found to be 0.725 (Figure 4J). Specifically, in the UHR group, 27 patients had a median PFS of 16.73 months, significantly reduced than 61.60 in the no‐UHR group, which included 96 patients with NDMM (HR = 3.412, *P*<0.001) (Figure 4F).

### 3.5 The +1q21‐involved UHR model potentially outperformed the R‐ISS and R2‐ISS in predicting the time to early relapse

Both R‐ISS stage 3 and R2‐ISS stage 4 usually indicate high‐risk progression displaying early relapse in clinical practice. In the training set, the median PFS was obviously shorter in the UHR group (15.00 months) than in the group at R‐ISS stage 3 (24.45 months, HR = 1.411, *p* = 0.169) and the group at R2‐ISS stage 4 (22.80 months, HR = 1.139, *p* = 0.621), although there were no statistical differences (Figure 4D). Similarly, in the validation set, the median PFS in the UHR group was 16.73 months, obviously shorter than the group at R2‐ISS stage 4 (22.27 months, HR = 1.720, *p* = 0.164) and significantly shorter than in the group at RISS stage 3 (23.57 months, HR = 1.990, *p* = 0.045) (Figure 4I).

## 4 DISCUSSION

Given that +1q21 is now included as a high‐risk factor in the latest R2‐ISS staging, it is crucial to elucidate its precise risk implications in the Chinese population, considering that nearly half of these patients harbor this marker.^21^, ^22^ Obviously, it is impractical to solely classify such a significant portion as high‐risk based solely on the presence of +1q21. Furthermore, the distinction of Gain (1q21) and Amp (1q21) in NDMM patients with +1q21 remains worthy of attention, contributing to the accurate interpretation on the risk role of +1q21 in a considerable population. In our model, weight scores were assigned to Gain (1q21) as 5.6 and Amp (1q21) as 9.7. This suggested that Amp (1q21) had a more pronounced adverse impact than Gain (1q21) on the prognosis of MM, consistent with previous studies.^13^, ^17^

In the study, the patients with Gain (1q21)‐combined and Amp (1q21)‐involved mutations exhibited a significantly inferior PFS compared to those without the +1q21 aberration, demonstrating that solely Amp (1q21), rather than solely Gain (1q21), are associated with poor outcomes in our center. It is consistent with the previous research,^23^ while it also revealed that among individuals with MGUS, Smoldering MM, and MM, the 10‐year PFS rates for those with 2, 3, and >3 copies of 1q21 were 72.2%, 42.5%, and 43.4%, respectively.^24^ Another study further underscored that solely Amp (1q21) was linked to a poorer prognosis.^25^ Moreover, a separate study encompassing 96 MM patients highlighted that those with Amp (1q21) exhibited a markedly lower 2‐year PFS rate, as compared to those with Gain (1q21) and those without +1q21, with rates standing at 23.3% versus 50.6% and 65.2%, respectively.^5^ Notably, the latest study reveals that small‐scale 1q amplification, though not significantly affecting patient survival, marks an early genetic event in 1q amplification, emphasizing the importance of regular cytogenetic monitoring to detect potential dynamic development and enable timely intervention.^26^

The study reaffirms the efficacy of ASCT, which is widely acknowledged as a standard consolidative therapy for NDMM,^27^ in overcoming the detrimental effects of +1q21 on survival outcomes.^11^, ^28^, ^29^ Additionally, another study demonstrated that ASCT significantly improved the overall response rate (ORR) and overall survival (OS) in patients with +1q21.^30^ A further investigation highlighted that upfront ASCT effectively mitigated the adverse prognostic impact in patients with Gain (1q21), though its benefits were not observed in those with Amp(1q21).^31^ It is noteworthy that the comparatively low adoption rate of ASCT in the study concurs with other reports from China,^31^, ^32^ an issue that is currently being actively addressed and enhanced in our region.

In this study, a risk scoring model based on +1q21 was proposed to predict early relapse in MM, especially for those with +1q21. This model encompasses 4 pivotal clinical parameters: Gain (1q21), Amp (1q21), TP53 deletion and no‐ASCT. It demonstrates superior predictive accuracy over the advanced staging in both the R‐ISS and R2‐ISS systems, especially in forecasting the timeframe to early relapse across both the training and validation cohorts. The TP53 biallelic inactivation (through deletion and/or mutation) and ISS stage 3 combined with Amp (1q21) have recently been identified as the two high‐risk factors.^33^ Another study revealed that the +1q21 marker identifies an exceptionally high‐risk population with a poor prognosis among patients with R‐ISS stage 3.^11^, ^14^, ^33^ This model can be applied to risk assessment for NDMM patients with varied copies numbers of +1q21, especially when making decisions about whether to perform ASCT. A salient aspect of this model lies in the significant influence of a no‐ASCT score of 8.5, highlighting the pivotal role of ASCT in whether stratifying patients into the UHR group with the total score ≥ 15.60 points. For instance, a patient who possessed both Gain(1q21) and TP53 deletion, resulting in a score of 13.1 when ASCT was taken into account, was originally classified in the UHR group. Nevertheless, without ASCT, the patient's classification would subsequently shift to the UHR group. Therefore, from another perspective, for myeloma patients with Amp(1q21) (9.7 points) who are ineligible for transplantation (8.5 points), they will be directly classified into the UHR group because their total score is greater than 15.60 points. This will indicate in advance that they should require more effective novel regimens, including the DRd regimen (daratumumab, lenalidomide, dexamethasone),^34^ the Isa‐VRd regimen (isatuximab, lenalidomide, bortezomib, dexamethasone)^35^ and so on.

The present study acknowledges several limitations, including its retrospective design, the variability in treatment regimens across the patient cohort, and the inherent constraints of being a single‐center investigation. A considerable proportion of patients in this study who did not undergo upfront ASCT, especially in line with other reports,^31^, ^32^ is currently benefiting from active efforts in China to increase patient awareness and expand eligibility platforms for transplantation. The primary reasons for the limited utilization of ASCT were patient age and frailty, with nearly 20% of the eligible population deterred by financial considerations.^31^ To enhance the credibility of our UHR model, which integrates Gain (1q21), Amp (1q21), the absence of ASCT, and TP53 deletion, further validation through external data sets is both necessary and planned.

## 5 CONCLUSION

Our research underscores the critical importance and operational algorithm of integrating +1q21 chromosomal abnormalities into risk assessment models. The UHR model, which incorporates both Gain (1q21) and Amp (1q21) alongside no‐ASCT and TP53 deletion, could serve as a valuable tool for identifying the truly functional HR subgroup among NDMM patients with +1q21. By doing so, healthcare providers can customize treatment approaches to not only potentially avert early relapse but also enhance long‐term survival outcomes.

## AUTHOR CONTRIBUTIONS

**Yanqiu Xiong:** Conceptualization (equal); data curation (equal); writing – original draft (equal). **Shanshan Liang:** Conceptualization (equal); data curation (equal); writing – original draft (equal). **Wenjiao Tang:** Data curation (equal). **Li Zhang:** Project administration (equal). **Yuhuan Zheng:** Investigation (equal); resources (equal). **Ling Pan:** Methodology (equal); visualization (equal). **Ting Niu:** Project administration (equal).

## FUNDING INFORMATION

This study was financially supported by the Key Research Project of Sichuan Anti‐Cancer Association Clinical Research (Grant No XH2023 5008).

## CONFLICT OF INTEREST STATEMENT

The author declares that there is no conflict of interest.

## ETHICS STATEMENT

The study was approved by the Institutional Ethics Committee of West China Hospital of Sichuan University and complied with the Declaration of Helsinki.

## Data Availability

The data that support the findings of this study are available from the corresponding author upon reasonable request.
